# Molecular epidemiology of human Borna disease virus 1 infection revisited

**DOI:** 10.1080/22221751.2022.2065931

**Published:** 2022-05-23

**Authors:** Liv Bode, Yujie Guo, Peng Xie

**Affiliations:** a Freelance Bornavirus Workgroup, Joint Senior Scientists, Berlin, Germany; b Department of Neurology, Yongchuan Hospital of Chongqing Medical University, Chongqing, People’s Republic of China; c NHC Key Laboratory of Diagnosis and Treatment on Brain Functional Diseases, The First Affiliated Hospital of Chongqing Medical University, Chongqing, People’s Republic of China; d Department of Neurology, The First Affiliated Hospital of Chongqing Medical University, Chongqing, People’s Republic of China

**Keywords:** Borna disease virus 1, BoDV-1, human complete BoDV-1 genomes, human and shrew amino acid BoDV-1 mutations, epidemiological concepts

## Abstract

Borna disease virus 1 (BoDV-1) strains attracted public interest by recently reported rare fatal encephalitis cases in Germany. Previously, human BoDV-1 infection was suggested to contribute to psychiatric diseases. Clinical outcomes (encephalitis vs. psychiatric disease) and epidemiology (zoonotic vs. human-to-human transmission) are still controversial. Here, phylogenetic analyses of 18 human and 4 laboratory strains revealed close genomic homologies both in distant geographical regions, and different clinical entities. Single unique amino acid mutations substantiated the authenticity of human strains. No matching was found with those of shrew strains in the same cluster 4, arguing against zoonosis. Opposite epidemiology concepts should be equally considered.

Borna disease virus 1 (BoDV-1) strains are key viruses of the species *Mammalian 1 orthobornavirus* within the ancient family *Bornaviridae*. Their non-segmented negative-strand RNA genome (8.9 kb, 6 proteins) replicates in the nucleus of infected cells [1]. BoDV-1 preferentially infects the limbic system of the brain and establishes persistent infections [2].

Human isolates were reported to be recovered from peripheral blood mononuclear cells (PBMCs) [3] and brain [4] of German and Japanese psychiatric patients. Whether a virus may contribute to mental disorders, triggered global research, but remained contraposing, despite global clues [5]. Unusually high genomic homology of BoDV-1 viruses (>95%) [1, 6] facilitated doubts about human isolates. They were contradicted by sequence identity between sample and isolate [7], and single amino acid (aa) mutations [3, 7] vs. laboratory strain V [1].

In 2018, BoDV-1 was shown to cause fatal human encephalitis in transplant recipients [8] and patients without impaired immune system [9, 10]. Retrospective analysis retrieved eight more cases (1999–2019), confirmed through next-generation sequencing [11]. Serology detected three BoDV-1 encephalitis cases out of 103 (2.9%) with unknown aetiology [12]. However, human BoDV-1 fatalities, in Bavaria [11] up to Northeast Germany [13], remained to be very rare.

BoDV-1 encephalitis reports used phylogenetic analyses (11.9% of the genome) to argue that human infections are both fatal and zoonotic, transmitted by bi-coloured, white-toothed shrews in endemic clusters in Southern Germany [11]. This concept became the leading opinion [8–13]. However, proofs either for shrew-to-human or human-to-human transmission, except transplant-related [8], were lacking.

Here, we re-evaluated available whole genomes of human strains. Furthermore, we analysed amino acid (aa) translates of human and shrew strains with high nucleotide homology.

Phylogenetic analyses, conducted in MEGA11 [14], compared 14 BoDV-1 genomes derived from encephalitis cases [8–13], four from PBMCs [6] and brain [4] of psychiatric patients, and four laboratory strains [1, 6]. To 21 published genomes we added the novel whole sequence of Hu-H1, now also accessible at GenBank (ON241315).

Ten strains each were assignable to either cluster- 4 or cluster- 1A, two strains to either cluster-2 or cluster-3 (Figure 1). In cluster- 4, strains from psychiatric patients (Hu-H1, Hu-H2; huP2Br, Hu-BV) [3, 7, 4] were joined together with encephalitis cases (112-16, ER-2, P3) [9–11]. However, their close genomic relationship (98%) (Table S1, supplement) corresponded neither to original locations (Germany, Japan), nor to years of isolation (1994–2016). Cluster-4 genomes were genetically distant from cluster-1A, cluster-2, and cluster-3 [8, 11–13]. Despite neighboured locations (Berlin and Brandenburg), BoDV-1 genomes belonged to distant cluster-4 and -3, respectively (Figure 1).

**Figure 1.**
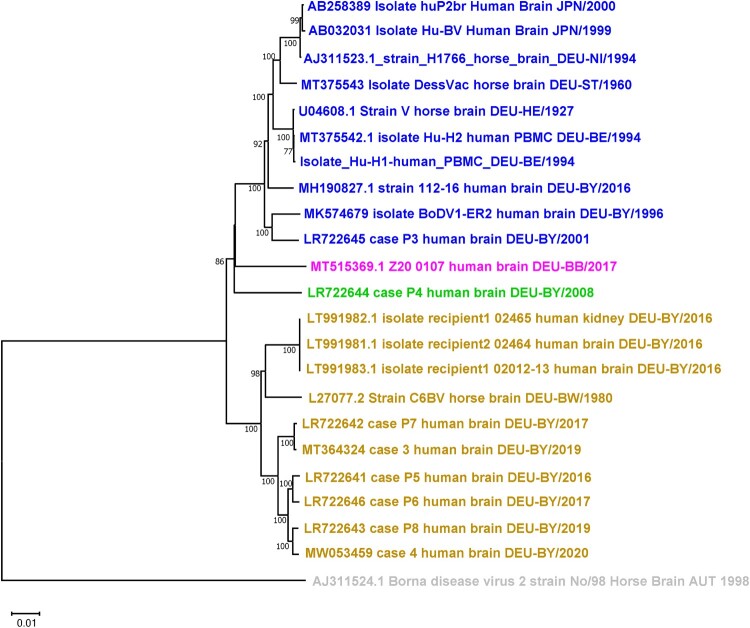
Phylogenetic tree of whole-genome sequences of BoDV-1 strains. Phylogenetic analysis used the Neighbour-Joining algorithm and p distance model in MEGA 11 [14]. The tree was rooted with the genome sequence of BoDV-2 No/98 (AJ311524). Values at branches represent support in 1000 bootstrap replicates. Only bootstrap values ≥70 at major branches were shown. Names indicated accession number at GenBank, description of isolate, original source, location, and year of isolation. Colour codes indicated designated cluster [11]; 1A = yellow; 2 = green; 3 = pink; 4 = blue. DEU = Germany; JPN = Japan; DEU Federal States: BE = Berlin; BB = Brandenburg; BW = Baden-Wurttemberg; BY = Bavaria; HE = Hesse; NI = Lower Saxony; ST = Saxony-Anhalt.

Laboratory strains (horse origin) belonged to either cluster-1A (C6BV, 1980) or cluster-4 [11], namely laboratory str. V (1927), which underwent multiple species changes until sequencing (1994) [1], strain H1766 (1994), and vaccine strain DessVac (Saxony-Anhalt, 1960) [6]. Cluster-4 laboratory strains displayed 98% homology between themselves and human strains, except a higher homology of Hu-H1 and Hu-H2 and laboratory str. V, and the Japanese strains and H1766 (99.9%) (Table S1, supplement).

Amino acid mutation analyses vs. reference str. V focused on cluster-4 strains [11]. We evaluated seven human and two laboratory strains on one part (Figure 2; Table S2 [supplement]), and seven shrew strains on the other part (Table S3; Table S4 [supplement]). Mutation percentages reflected low levels of divergence for N-, M-, and P-proteins (<1%), a moderate level for the large polymerase (L-Pol) (<2%), and high levels for the G-protein (2%-4%) and the non-structural X-protein (5%-10%). Human strains not only displayed 1.6-fold more changed amino acids than shrews (*N* = 63 vs. 39). Almost half of these changes (29/63 = 46.0%) were unique as compared to only a quarter (10/39 = 25.6%) in shrews, and mainly non-conservative in both hosts (≥ 60%).

**Figure 2.**
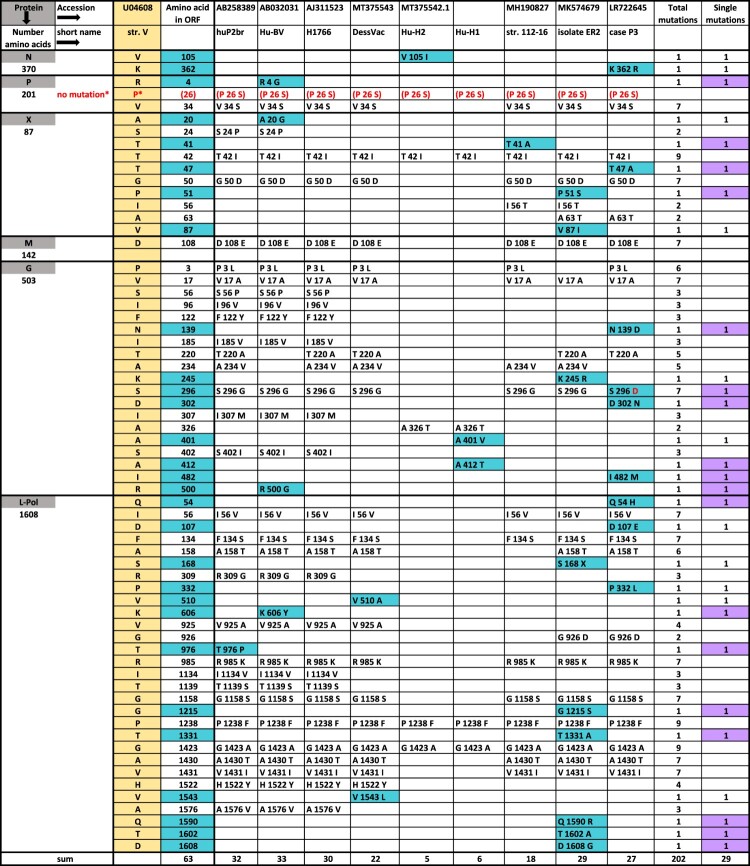
Evaluation of amino acid mutations of BoDV-1 strains. Amino acid (aa) changes of seven human and two laboratory cluster-4 viruses vs. strain V using the one-letter-code. Background colour in turquoise indicates all single changes, in lilac non-conservative single changes. *P indicates a mistake in sequence U04608. P 26 S is the correct reading. S26/S28 is the major phosphorylation site of P-protein. For aa alignments, see Table S2.

Notably, none of the 10 unique shrew mutations (Table S3, supplement) were matching with any of those in human strains. Each strain, either of human or shrew origin, differed by an individual aa-mutation pattern, despite 98% nucleotide homology. For example, encephalitis cases ER2 [10] and P3 [11], both located in Northern Bavaria, shared none of their nine unique aa mutations. Very few mutations (two each in G- and L-pol protein), were previously shown to elicit enhanced neurovirulence in rats [15]. Similarly, Hu-H1 and Hu-H2, isolated from PBMCs of a patient with bipolar depression and obsessive-compulsive disorder, respectively [3], promoted apoptosis and inhibited cell proliferation, contrasting opposite effects by str. V [16, 6], despite differing by only few aa- mutations. The Japanese strains, huP2br, isolated from the brain of a schizophrenic patient [4], and Hu-BV differed from each other and H1766 by five unique mutations (Figure 2).

## Conclusion

First, human BoDV-1 genome analyses confirmed sequence similarities between distant geographic regions [6] suggesting global prevalence [5] rather than narrow endemic areas [8–13]. Second, strains in cluster-4 indirectly suggested a broad clinical spectrum. Third, amino acid analyses demonstrated the authenticity of human strains by individual mutation signatures. Fourth, no match occurred between unique aa-mutations of shrews and those of human strains, arguing against zoonosis. Fifth, opposite epidemiology concepts should be considered equally, namely zoonotic versus human-to-human transmission driven by unnoticed healthy carriers [5].

## Supplementary Material

TEMI_2065931_Supplemental_Material
